# Identification of Human Monoclonal Antibodies Specific for Human SOD1 Recognizing Distinct Epitopes and Forms of SOD1

**DOI:** 10.1371/journal.pone.0061210

**Published:** 2013-04-17

**Authors:** Teresa J. Broering, Hongyan Wang, Naomi K. Boatright, Yang Wang, Katherine Baptista, Gilda Shayan, Kerry A. Garrity, Can Kayatekin, Daryl A. Bosco, C. Robert Matthews, Donna M. Ambrosino, Zuoshang Xu, Gregory J. Babcock

**Affiliations:** 1 MassBiologics, University of Massachusetts Medical School, Boston, Massachusetts, United States of America; 2 Department of Biochemistry and Molecular Pharmacology, University of Massachusetts Medical School, Worcester, Massachusetts, United States of America; 3 Department of Neurology, University of Massachusetts Medical School, Worcester, Massachusetts, United States of America; 4 Department of Cell Biology, University of Massachusetts Medical School, Worcester, Massachusetts, United States of America; 5 Neuroscience Program, University of Massachusetts Medical School, Worcester, Massachusetts, United States of America; Louisiana State University Health Sciences Center, United States of America

## Abstract

Mutations in the gene encoding human SOD1 (hSOD1) can cause amyotrophic lateral sclerosis (ALS) yet the mechanism by which mutant SOD1 can induce ALS is not fully understood. There is currently no cure for ALS or treatment that significantly reduces symptoms or progression. To develop tools to understand the protein conformations present in mutant SOD1-induced ALS and as possible immunotherapy, we isolated and characterized eleven unique human monoclonal antibodies specific for hSOD1. Among these, five recognized distinct linear epitopes on hSOD1 that were not available in the properly-folded protein but were available on forms of protein with some degree of misfolding. The other six antibodies recognized conformation-dependent epitopes that were present in the properly-folded protein with two different recognition profiles: three could bind hSOD1 dimer or monomer and the other three were specific for hSOD1 dimer only. Antibodies with the capacity to bind hSOD1 monomer were able to prevent increased hydrophobicity when mutant hSOD1 was exposed to increased temperature and EDTA, suggesting that the antibodies stabilized the native structure of hSOD1. Two antibodies were tested in a G93A mutant hSOD1 transgenic mouse model of ALS but did not yield a statistically significant increase in overall survival. It may be that the two antibodies selected for testing in the mouse model were not effective for therapy or that the model and/or route of administration were not optimal to produce a therapeutic effect. Therefore, additional testing will be required to determine therapeutic potential for SOD1 mutant ALS and potentially some subset of sporadic ALS.

## Introduction

Amyotrophic lateral sclerosis (ALS), also known as Lou Gehrig’s disease, is characterized by progressive motor neuron degeneration, muscle wasting and paralysis [1]. There is currently no cure and paralysis progressively proceeds from loss of gross motor control to loss of breathing capacity and ultimately death. Motor neurons are selectively affected with cognitive function largely retained. Current treatments consist primarily of supportive care and one approved drug Riluzole, which provides a modest extension of life of approximately three months [2].

Of patients diagnosed with ALS, approximately 10% have a family history of the disease (familial ALS or fALS), and the other 90% have no known family history (sporadic ALS or sALS). Mutations in multiple genes have been associated with fALS, and the gene encoding Cu/Zn superoxide dismutase 1 (SOD1) has mutations in approximately 20% of fALS cases, ranking second in frequency among currently identified gene mutations [3]–[5]. Symptoms of sALS and fALS are clinically indistinguishable suggesting that there may be common pathways involved in both forms of the disease [6]. Recent work suggests that oxidized or misfolded SOD1 can be found in some but not all sALS patients [7]–[10]. Thus, misfolded SOD1 could be involved in disease pathogenesis in both fALS and sALS patients.

SOD1 is ubiquitously expressed in the cytoplasm with high levels in motor neurons. The 32 kDa SOD1 homodimer contains two molecules of both copper and zinc with an intramolecular disulfide bond present in each monomer [11]. There are over 150 different identified mutations in the 153 amino acid human SOD1 protein (hSOD1) that are associated with fALS (http://alsod.iop.kcl.ac.uk/als). Mutant hSOD1 protein expression has many documented effects on cells: disruption of axonal transport [7], interference with mitochondrial function [12], inclusion formation [13], atypical secretion of hSOD1 from cells [14], and others. However, the mechanisms of disease pathology and symptoms caused by mutant hSOD1 are not fully understood [15]. A common effect of various mutations in hSOD1 is decreased SOD1 stability and an increased propensity of SOD1 to misfold and aggregate [16]. It is proposed that misfolded SOD1 may directly or indirectly cause motor neuron death.

Several well-established transgenic mouse models expressing different mutant hSOD1 proteins display the hallmarks of ALS [17]–[19]. Mice expressing mutant hSOD1 develop progressive paralysis that proceeds to an early death with evidence of motor neuron loss. This type of rodent model has been used to test numerous different compounds, but translation of treatments from the mouse model to human therapeutics has proven difficult [20]. To date, none of the many compounds tested have provided benefit to the human population with the exception of Riluzole which showed effects in rodent models of disease that were very modest and similar to many other compounds [21], [22]. The lack of correlation may be due to a multitude of factors including variation in the animal models and mutations in hSOD1 representing a small percentage of the total human ALS population.

Previous data from immunization and passive antibody transfer to mutant hSOD1 transgenic mice has provided an extension in survival in some mouse models. Immunization of G37R hSOD1 transgenic mice with mutant hSOD1 or immunization of low-copy G93A transgenic mice with mutant or wild-type hSOD1 lacking copper and zinc (apo-hSOD1) led to a delay in disease endpoint of 30 days and 14 days respectively [23], [24]. Similar immunizations in high-copy G93A transgenic mice did not result in a statistically significant delay of disease endpoint [23], [24]. hSOD1 dimer interface (SEDI) peptide immunization of G37R hSOD1 transgenic mice delayed disease endpoint 40 days [25]. The same peptide immunization in the high-copy G93A transgenic mouse model delayed disease endpoint 7 days but was not statistically significant [25]. Passive transfer of a proportion of mouse antibodies specific for hSOD1 also gave a small (6 days) but statistically significant delay in disease endpoint in the high-copy G93A hSOD1 mouse model [23], [26]. These results suggest that antibodies specific to mutant or misfolded hSOD1 may be an effective therapeutic for fALS with SOD1 mutation and potentially some cases of sALS.

To further pursue potential immunotherapy for fALS with SOD1 mutation, we generated a panel of fully human monoclonal antibodies (HuMabs) directed against hSOD1 to allow direct translation to treatment in affected individuals. The isolated HuMabs recognized various epitopes of hSOD1, and some HuMabs specifically recognized unfolded or misfolded but not properly-folded hSOD1. A select number of HuMabs were tested in a hSOD1 mutant transgenic mouse model. This panel of HuMabs should prove useful to probe the structure of various forms of hSOD1 in ALS and may have potential as a therapeutic.

## Materials and Methods

### Cells and Cell Culture

HEK-293T/17 and P3X-AG8.653 cells were obtained from the American Type Culture Collection (ATCC). HEK-293T/17 cells were grown in Dulbecco’s modified Eagle’s media (DMEM) supplemented with 10% fetal bovine serum (FBS) and 100 IU penicillin-streptomycin. P3X-AG8.653 cells were grown in a supplemented RPMI media and hybridomas were cultured in a DMEM supplemented media as previously described [27]. All cells were grown at 37°C in air supplemented with 5% CO_2_.

### Expression and Purification of Human SOD1 Fusion Proteins from Bacteria

A bacterially codon-optimized gene encoding the wild-type (WT) hSOD1 protein (UniProtKB/Swiss-Prot accession number P00441) was synthesized by Integrated DNA Technologies (IDT) and the sequence was confirmed. To express this protein in bacteria with an amino-terminal (N-terminal) thioredoxin fusion and carboxy-terminal (C-terminal) 6-histidine tag, the gene was subcloned from the vector provided from IDT into pET32a(+) (EMD) in frame with the upstream thioredoxin (Trx) gene and downstream 6-histidine tag using BamHI and SalI. The Trx N-terminal fusion led to increased solubility and higher expression levels allowing large amounts of protein production for use in the initial screening assays. To express hSOD1 in bacteria with an N-terminal glutathione sulfur transferase (GST) fusion, the gene was subcloned from the vector provided from IDT into pGEX-4T-3 (GE Healthcare) in frame with the upstream GST gene employing BamHI and SalI. BL21Star *Escherichia coli* cells (Invitrogen) were transformed with pET32a(+) and pGEX-4T-3 vectors and grown overnight at 37°C in Luria-Bertani broth containing 100 µg/ml ampicillin (LB-amp). The culture was diluted 1∶10 in LB-amp broth and grown for 2.5 hours at 37°C followed by addition of 1 mM isopropyl-ß-d-thiogalactopyranoside and further grown at 37°C for 2.5 hours. Bacteria were harvested by centrifugation and pellets were frozen at -20°C. Bacteria were lysed and proteins purified as described previously [28] employing Ni-NTA agarose for pET32a(+) vectors and glutathione sepharose for pGEX-4T-3 vectors. The human SOD1 expressed from bacteria with a Trx and 6-histidine tag is referred to as Trx-hSOD1-WT-his and the glutathione fusion is referred to as GST-hSOD1-WT. Mutagenesis of the bacterially codon optimized hSOD1 in the pET32a(+) expression plasmid was performed using the Quick Change II Site-Directed Mutagenesis kit (Stratagene) following the manufacturer’s instructions to introduce the following mutations into the hSOD1 gene: A4V, G93A, and G85R. DNA encoding fragments of SOD1 were amplified from pET-32a-hSOD1 using various sequence specific oligonucleotide primers and cloned into pET32a(+) in frame with the C-terminal 6-histidine tag. All constructs were confirmed by DNA sequencing. The hSOD1 mutants and fragments were expressed and purified as described above for the full-length Trx-hSOD1-WT-his protein.

### Mouse Immunizations and Hybridoma Isolation

Transgenic mice comprising unrearranged human immunoglobulin genes and inactivated mouse heavy and kappa-light chain loci [29] (provided by Medarex, Inc., a wholly owned subsidiary of Bristol-Myers Squibb Company) were injected weekly for a total of 7–20 weeks with 100 µg of various hSOD1 proteins mixed with Sigma adjuvant system (Sigma). This study was performed in accordance with the recommendations in the Guide for the Care and Use of Laboratory Animals of the National Institutes of Health. The protocol was approved by the Institutional Animal Care and Use Committee at the University of Massachusetts Medical School (protocol A-1780). Mammalian hSOD1 protein purified from human erythrocytes was purchased from Sigma (E-hSOD1) and has been previously noted to have post-translational modifications [30]. ELISA was employed to measure serum responses to antigen and animals were sacrificed when serum responses reached a plateau. Hybridomas were generated by the standard polyethylene glycol (PEG) method using P3X63-AG8.653 mouse myeloma cells as fusion partner.

### ELISA

Culture supernatants or purified antibody were assessed for antigen binding using ELISA. Microtiter plates (96 well) were coated with 100 µl per well of 0.5–2 µg/ml of antigen in phosphate buffered saline, pH 7.4 (PBS) overnight at 4°C. Antibody (100 µl) at varying dilutions was added and incubated at room temperature for 1 hr. Antibody binding was detected using an anti-human IgG-alkaline phosphatase (AP) conjugate (1∶5000, Jackson Immunoresearch) followed by p-nitrophenyl phosphate disodium salt (PNPP) at 1 mg/ml in 1 M diethanolamine. Absorbance (405 nm) was analyzed using Molecular Devices Emax plate reader with the Softmax software. For peptide specific ELISAs, overlapping peptides (New England Peptide) containing N-terminal long chain biotin were bound to streptavidin coated plates (Nunc) by incubating 100 µl per well of 1 µg/ml of peptide in PBS +0.05% Tween-20 at room temperature for 1 hr. The plates were then washed and antibody binding to peptides was detected using identical reagents as described above.

### Antibody Cloning, Expression, and Purification

Heavy and light chain variable regions were amplified from hybridoma RNA as described previously [27]. Heavy chain variable region PCR products were cloned into a mammalian expression vector in frame with human IgG1 constant region contained in the vector. Amplified light chain variable regions were cloned into a mammalian expression vector in frame with the human kappa constant region present in the vector. Heavy and light chain vectors were combined to a single vector and electroporated into CHO cells. Stable transfectants were selected and expanded. Hybridoma cell cultures or stably transfected CHO cells were expanded and supernatant harvested by centrifugation. Antibody was purified with protein A or G sepharose with acid elution followed by dialysis in PBS as described previously [27].

### Relative Avidity Determination and Competition Experiments

Anti-human biosensors (ForteBio) were used to capture HuMabs in PBS (10 µg/ml) followed by binding to various concentrations of hSOD1 proteins in PBS. Results for association and dissociation rate constants were calculated and used to derive the dissociation constant (K_D_) using the Octet and associated software (ForteBio). For competitive binding experiments, the first HuMab in PBS (10 µg/ml) was captured on an anti-human biosensor followed by binding to hSOD1 protein in PBS (137 nM). The biosensor was then transferred to the first HuMab solution to ensure that all antibody binding sites were saturated on the hSOD1 dimer. Binding was assessed for the second HuMab after introduction of the biosensor-HuMab-hSOD1 complex into a solution of the second HuMab in PBS (10 µg/ml).

### Generation of apo-hSOD1 and apo-hSOD1-monomer

A hSOD1 variant with the 2 free cysteines mutated (C6A/C111S) to preclude intramolecular disulfide-bond interchange was expressed in bacteria without fusion proteins or other tags from the pET3d vector in BL21-Gold (DE3) *Escherichia coli* and purified as previously described [31]. Apo-hSOD1 was generated by demetallation as described previously [32]. The apo-hSOD1 monomer was generated by introducing 2 additional mutations at the subunit interface (C6A/C111S/F50E/G51E) and was purified and demetallated.

### Transient Transfection of Human SOD1 WT and Point Mutants in Human Cells Followed by Immunoprecipitation

Mammalian expression vector (pcDNA, Invitrogen) encoding hSOD1 wild type (WT) and engineered mutants (A4V, G85R, and G93A) with a C-terminal myc tag were previously described [33]. HEK-293T/17 cells were transfected with expression vectors using lipofectamine 2000 (Invitrogen) as described by the manufacturer. Cells were harvested with PBS +5 mM EDTA on day 2 after transfection and lysed with PBS +0.1% Triton X-100 and complete protease inhibitors (Roche). The insoluble material was removed by centrifugation for 10 min at 14,000 rcf. Soluble material was immunoprecipitated with 2 µg of antibody and 50 µl of protein A sepharose beads. Samples were subjected to SDS-PAGE and immunoblotting with myc tag antibody (9E10) as a detection agent followed by anti-mouse horseradish peroxidase conjugate. Immunoblots were developed with chemiluminescent detection.

### Measurement of Increased Hydrophobicity Following Heating and EDTA Treatment of SOD1

To introduce a convenient protease cleavage site for fusion protein removal, the sequence encoding hSOD1 containing G85R or G93A point mutants was removed from pGEX-4T-3 with BamHI and SalI and ligated to pGEX-6P-1 (GE Healthcare) cut with the same enzymes. GST-hSOD1-G85R and GST-hSOD1-G93A were expressed and purified with glutathione beads as described above for GST-hSOD1-WT. The proteins were cleaved with PreScission protease (GE Healthcare) according to the manufacturer protocol. GST, PreScission protease and uncleaved GST-hSOD1 were removed with glutathione beads. The resulting hSOD1-G85R and hSOD1-G93A proteins each contained an additional six amino acids at the N-terminus (GPLGSM) after protease cleavage and had a native C-terminus. For the hydrophobicity assay, equimolar amounts of hSOD1 mutant protein and HuMab, along with 5 mM EDTA (final concentration) were co-incubated at room temperature or at 45°C for 4 hours (this corresponded to 40 µg of hSOD1 with 200 µg of HuMab in a final volume of 30 µl). Samples were then cooled to room temperature and placed in an opaque, black 96-well plate. To each well, 8-anilino-1-naphthalenesulfonic acid ammonium salt (ANS, Fisher Scientific) was added to a final concentration of 20 µM and incubated at room temperature for 30 minutes. The fluorescence was measured on a Victor 3 plate reader (Perkin Elmer) with an excitation wavelength of 390 nm and emission of 460 nm.

### Mutant hSOD1 Mouse Model

Transgenic mice for hSOD1-G93A (B6SJL-Tg(SOD1G93A)1Gur/J strain) were obtained from Jackson Laboratories. These studies were performed in accordance with the recommendations in the Guide for the Care and Use of Laboratory Animals of the National Institutes of Health. The mouse experiments were performed with approval from the Institutional Animal Care and Use Committee at University of Massachusetts Medical School under protocols A-1690 and A-2250. Only male mice were used to reduce gender differences in disease onset and progression. For intrathecal (IT) dosing, HuMabs was delivered over 50 days starting at day 65 from an Alzet 2006 osmotic pump (DURECT corp.) with an attached catheter implanted into the mouse lumbar subarachnoid space between the L5 and L6 vertebra as previously described [34]. Mice had to reach or exceed a weight of 20 g prior to pump implantation so day 65 was chosen because of mouse size and allowing approximately 20 to 35 days of HuMab dosing prior to symptom onset. We chose to use the longest duration pump available for mice. The pump was primed with each HuMab (240 µl of 10 to 16 mg/ml for a total 2.4 to 3.8 mg) 24 to 48 hr prior to implantation and delivered 0.2 µl/hr for 50 days. After 50 days at day 115 when the pump had completed the programmed delivery duration, the pump and catheter were removed with a second survival surgery. Any remaining HuMab was collected from the pump after removal to determine HuMab concentration and activity. The end stage of disease was defined as the day of complete paralysis of two limbs as judged by a researcher blinded to the treatment groups. At disease endpoint, mice were anesthetized and sera and spinal cord tissue collected. Mice were perfused with PBS prior to spinal cord tissue removal. Mice were weighed 2–3 times per week throughout the course of the experiment. For intraperitoneal (IP) dosing, each HuMab was given via IP injection starting at day 65 (0.2 ml of 6.25 mg/ml in PBS, 50 mg/kg final dose) for 3 consecutive days at initial dosing and then once every 7 days until disease endpoint was reached. Mice were observed for disease endpoint and spinal cord tissue collected from a subset of the mice without perfusion. Tissue was washed briefly in PBS prior to freezing. Survival analysis was performed using JMP software (SAS) with Mantel-Cox (Log-Rank) test for probability.

## Results

### Antibody Generation and Selection for Further Characterization

To generate a panel of human monoclonal antibodies (HuMabs) directed against the human SOD1 (hSOD1) protein, we immunized mice transgenic for human immunoglobulin heavy and light chain genes [29] (provided by Medarex Inc., a wholly owned subsidiary of Bristol-Myers Squibb) with various hSOD1 antigens. Spleens from a total of seventeen mice were fused, and screening with an antigen-specific human IgG ELISA yielded 879 hybridomas reactive to bacterially-produced thioredoxin-hSOD1 fusion protein (Trx-hSOD1-WT-his). To select a unique panel of HuMabs, we determined and compared the sequences of the antibody heavy chains, the affinities for Trx-hSOD1-WT-his, and the antibody epitopes (described in detail in subsequent sections). Eleven HuMabs were chosen for complete characterization by selecting a representative antibody from each unique heavy chain sequence that had an avidity for Trx-hSOD1-WT-his that was 20 nM or lower. An additional selection criterion for the antibodies was the capacity to bind both hSOD1 with a G93A mutation (present in our chosen mouse model) and hSOD1 with an A4V mutation (the most prevalent human mutation in the North America) [6]. The eleven HuMabs were derived from eight different mice immunized with a variety of different hSOD1 proteins (Table S1 in File S1). The antibodies represented various V_H_ and V_κ_ families and contained unique complementarity determining regions (Tables S2 and S3 in File S1).

To produce the quantities of antibody required for our studies, the heavy and light chain genes of the selected HuMabs were cloned into mammalian expression vectors and expressed in CHO cells. Of note, the HuMab 37 constant region was IgG3 from the hybridoma but was converted to an IgG1 constant region when cloned into a mammalian expression vector. Relative avidity for hSOD1 of cloned HuMab 37 in the context of an IgG1 heavy chain was essentially identical to that of the IgG3 HuMab 37 produced from the isolated hybridoma (data not shown). Identical epitope mapping results were obtained with both isotype versions of the antibody as well (data not shown).

### Identification of Linear and Conformation-dependent Epitopes on hSOD1

**Figure 1:**
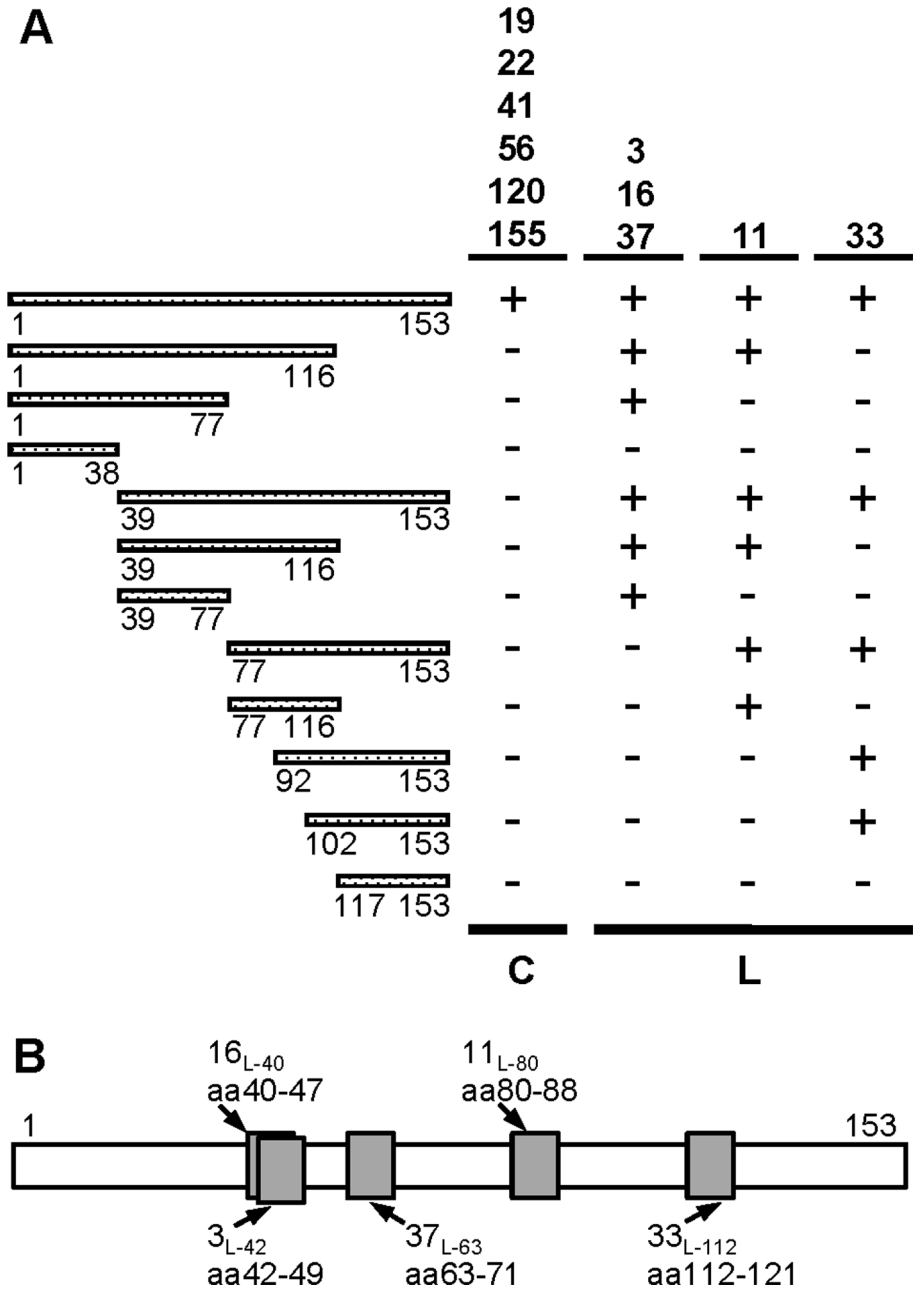
HuMabs recognize different regions of the hSOD1 protein. (A) Full-length hSOD1 (amino acids 1–153) and various portions of the protein were expressed in bacteria fused to the carboxy-terminus (C-terminus) of thioredoxin (Trx) and containing a C-terminal 6-histidine tag used for purification (Trx-hSOD1-WT-his). Each protein is represented in the figure as a hashed line with the beginning and ending amino acid number listed below the line. The proteins were coated on ELISA plates and binding of HuMabs (listed at the top right) detected with goat-anti-human antibody conjugated to alkaline phosphatase followed by PNPP substrate addition. ELISA results are listed to the right of the schematic; positive recognition is indicated by a plus sign while signals equivalent to background are indicated by a minus sign. HuMabs recognizing only full-length hSOD1 were designated conformation dependent and are noted below with a C. HuMabs with an epitope that mapped to a linear sequence of amino acids are noted below with an L. (B) Minimal linear epitopes were determined with amino-terminal biotin-labeled overlapping peptides coated on streptavidin ELISA plates. Binding of HuMabs was assessed as described in A. The epitopes are noted as a grey box for each linear-epitope HuMab with the amino acids (aa) bound indicated. To distinguish these epitopes throughout the rest of the manuscript each linear-epitope HuMab has a subscripted L for linear followed by the initial amino acid of the epitope.

To map the amino acids of hSOD1 bound by each HuMab, we cloned, expressed and purified protein fragments of hSOD1 and screened for antibody recognition. hSOD1 was divided into four roughly equal sections which were generated in bacteria as a fusion protein to thioredoxin (Trx) to increase solubility and expression levels. The fusion proteins also had a C-terminal 6-histidine tag to aid in purification. Progressive truncations of the protein were also made from each terminus, and the central portion of the protein was expressed to provide overlap if an epitope fell across the arbitrary divisions between the four major truncations (Figure 1A). The truncated proteins and full-length bacterial Trx-hSOD1-WT-his were used in an ELISA-based screen with HuMabs as detection agents. A 6-histidine tag antibody was used as a control to ensure that all bacterial proteins were available for antibody recognition (data not shown). Six of the hSOD1-specific HuMabs (19, 22, 41, 56, 120, and 155) gave no detectable signal above background with the truncations and only recognized the full-length protein (summarized in Figure 1A). The binding of these six HuMabs was designated as conformation-dependent since truncation at different ends of the protein caused a loss of binding suggesting that the overall native conformation of the protein was lost. To distinguish these HuMabs throughout the rest of this manuscript, they will be noted with a subscript of ‘c’ for conformation-dependent (e.g., 41_c_). The other five HuMabs were each found to bind a linear sequence of amino acids. HuMabs 3, 16, and 37 recognized amino acids 39–77 as a minimal binding domain (Figure 1A). HuMab 11 bound to amino acids 77–116, and HuMab 33 recognized amino acids 102-153 (Figure 1A). To further define the epitopes and determine if HuMabs 3, 16, and 37 bound distinct regions of the hSOD1 protein, a panel of overlapping peptides was screened for binding in an ELISA. Each of the HuMabs recognized a distinct set of peptides with an epitope of ten amino acids or less and thus each was designated as binding a linear epitope (data not shown). Two of the HuMabs, 3 and 16, had overlapping epitopes consisting of amino acids 42–49 and 40–47, respectively (summarized in Figure 1B). HuMab 37 had a distinct epitope of amino acids 63–71. The epitope identified for HuMab 11 was amino acids 80–88 and for HuMab 33 was amino acids 112–121. To distinguish the HuMabs that bind linear epitopes throughout the rest of the manuscript, each HuMab number will have a subscript with an ‘L’ for linear, followed by the initial amino acid number of the determined minimal epitope (e.g., 16_L-40_).

### Competition of HuMabs for hSOD1 Binding

**Figure 2:**
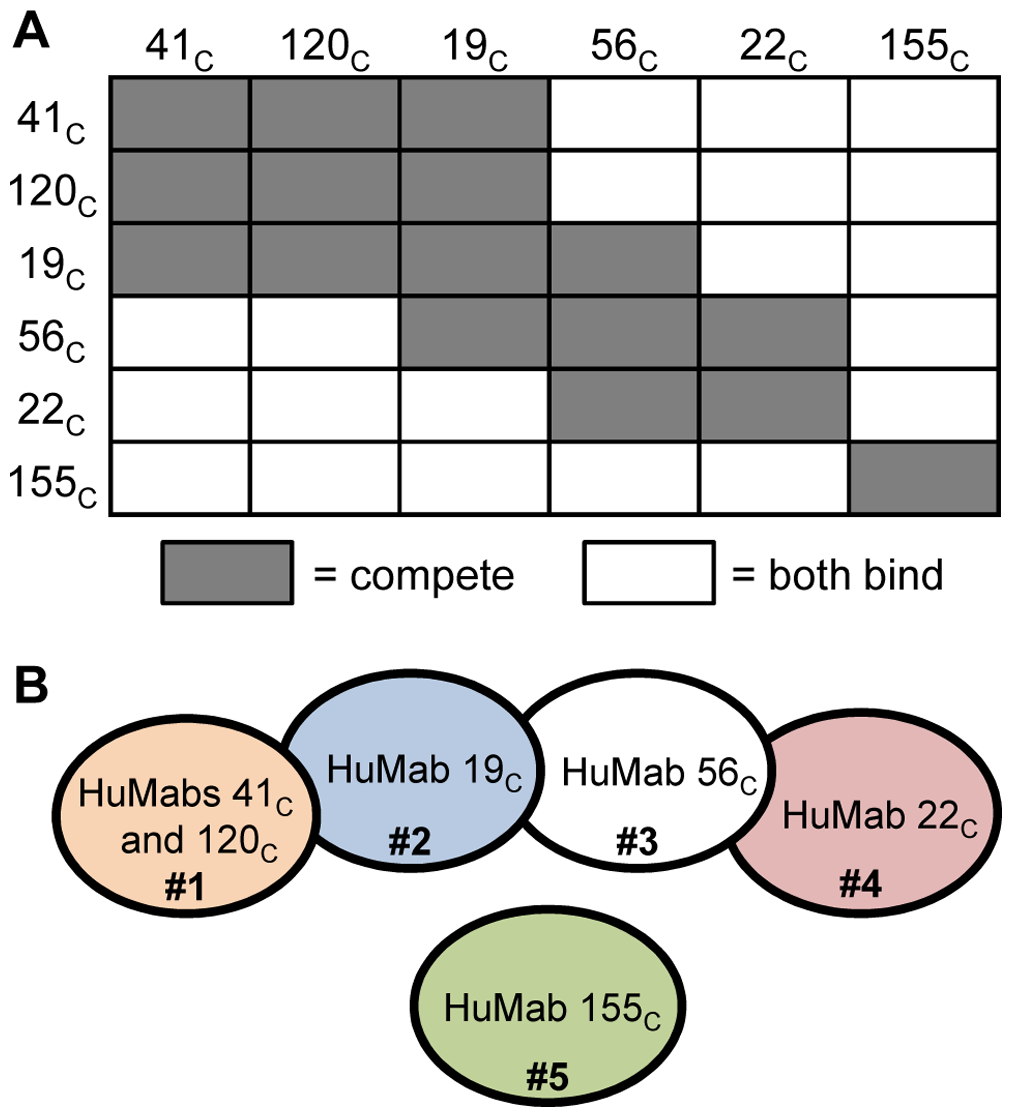
Competition of various HuMabs for binding to Trx-hSOD1-WT-his. (A) HuMabs designated as conformation-dependent where assayed for simultaneous binding of hSOD1. The HuMab listed to the left of the graph was bound to an anti-human biosensor and then allowed to bind Trx-hSOD1-WT-his. The second antibody (listed at the top of the graph) was then assayed for binding to the biosensor through interaction with Trx-hSOD1-WT-his. Simultaneous binding of both antibodies is indicated as a white box. Competing antibodies are indicated as a dark grey box. (B) The epitopes of the conformation-dependent HuMabs are represented graphically by overlapping circles. Circles that do not overlap represent antibodies that can bind the protein simultaneously. Circles that overlap are competing epitopes. Based on this profile, the conformation-dependent epitopes were grouped into five different binding profiles numbered from one to five.

To determine if the HuMabs designated as conformation-dependent bound to distinct or competing epitopes on the hSOD1 protein, an assay was developed to assess simultaneous binding of two antibodies to the Trx-hSOD1-WT-his protein. This assay designated the epitopes as distinct if both antibodies could bind the protein simultaneously or designated the epitopes as competing if the binding of the first antibody to hSOD1 prevented the binding of the second antibody. Each combination of the six conformation-dependent HuMabs was assayed including swapping the order of antibody binding. An isotype-matched irrelevant antibody was included, as well as, controls that lacked antibody or hSOD1 protein. No binding to Trx-hSOD1-WT-his was demonstrated with either the irrelevant antibody or the no antibody controls (data not shown). If Trx-hSOD1-WT-his was not included in the assay, the second antibody was unable to bind indicating that additional antibody binding was mediated through hSOD1 (data not shown). The results for the competition analysis are summarized in Figure 2A with the HuMab bound to Trx-hSOD1-WT-his first listed to the left of the chart and the HuMab antibody which was assessed as the second antibody listed across the top of the chart. Failure of the second antibody to bind is designated as competing epitopes in dark gray. Simultaneous binding of the two antibodies is designated as distinct epitopes in white. Each HuMab competed for binding with itself ensuring that the binding was saturating for each antibody used (Figure 2A). The results are summarized in a graphic epitope map with overlapping circles representing competing epitopes and circles that do not touch representing HuMabs that can simultaneously bind to the Trx-hSOD1-WT-his protein (Figure 2B). The epitope for HuMabs 41_c_ and 120_c_ are similar and may be identical while the other conformation-dependent HuMabs have unique competition profiles. The binding of 155_c_ did not prevent the binding of other conformation-dependent HuMabs suggesting that the epitope is distinct from the other epitopes. This competition panel was repeated using commercially-available, natively-expressed mammalian hSOD1 purified from human erythrocytes (E-hSOD1) rather than the Trx fusion protein expressed in bacteria. Identical results were obtained suggesting that the conformation-dependent HuMabs bind to the E-hSOD1 and Trx-hSOD1-WT-his proteins in a similar manner (data not shown).

The competition assay was repeated including HuMabs with both linear and conformation-dependent epitopes binding to Trx-hSOD1-WT-his. The linear-epitope HuMabs gave the expected competition profile with each other based on the mapped epitopes. HuMabs 16_L-40_ and 3_L-42_ competed for binding to Trx-hSOD1-WT-his while the other HuMabs linear epitopes were distinct and able to bind the protein simultaneously. The exception was the inability of HuMab 3_L-42_ to bind if 37_L-63_ or 11_L-80_ were pre-bound (data not shown). Interestingly, each of the five linear-epitope HuMabs competed for binding to Trx-hSOD1-WT-his with each of the six conformation-dependent HuMabs (data not shown). This was the case whether the linear-epitope HuMab was bound first or second. It is possible that each of the five different conformation-dependent epitopes physically overlap with each of the four linear epitopes. However, given that both conformation-dependent and linear-epitope HuMabs fall into multiple non-competing groups, a more likely possibility is that linear and conformation-dependent HuMabs bind different conformations of hSOD1 and lock the protein into that conformation preventing the other type of antibody from binding.

As done with the conformation-dependent HuMabs, the linear-epitope HuMab competition panel was repeated with the mammalian-expressed E-hSOD1. We found no detectable binding for the linear-epitope HuMabs to E-hSOD1 (see next section for further detail). The conformation-dependent HuMabs were still able to bind to E-hSOD1 with the same competition profile seen in Figure 2A. These results suggest that the conformation of E-hSOD1 is different than that of Trx-hSOD1-WT-his and does not allow binding of the linear-epitope HuMabs.

### HuMab Avidity for Various Forms of hSOD1

To further explore the binding differences found for the linear-epitope HuMabs, we determined avidity measurements of the HuMab panel for various forms of the hSOD1 protein. The hSOD1-specific HuMabs were originally selected to bind Trx-hSOD1-WT-his generated from bacterial expression and purification without additional copper or zinc. Analysis of this protein with inductively coupled mass spectrometry gave 1.54 zinc molecules and 0.09 copper molecules per dimer of Trx-hSOD1-WT-his. Simultaneous analysis of E-hSOD1 gave 0.82 zinc molecules and 0.83 copper molecules per dimer. The differing metal content of the two proteins or the presence of a fusion protein could alter the conformation of the SOD1 protein. Thus, we used avidity determination to make relative comparisons of the binding of the HuMabs to Trx-hSOD1-WT-his and E-hSOD1. Antibody was captured on an anti-human IgG biosensor (ForteBio) and the association and disassociation rates were determined using the Octet and associated software. The calculated affinity constant (K_D_) for each HuMab is reported in Table 1. Each HuMab had a high avidity for the Trx-hSOD1-WT-his protein with K_D_ from 1 to 7 nM. The K_D_ of the conformation-dependent HuMabs for the E-hSOD1 protein were similar to constants calculated for Trx-hSOD1-WT-his (Table 1). In contrast, K_D_ could not be calculated for the linear-epitope HuMabs for E-hSOD1 as no binding was detected between these five HuMabs and this mammalian-produced hSOD1 (Table 1). Similar results were obtained when binding was assessed with a capture ELISA coating HuMabs on the plate and detecting bound hSOD1 with a polyclonal antibody (data not shown).

**Table 1:** HuMab avidity for various forms of hSOD1.

		K_D_ (nM)			
Antibody^a^	Epitope^b^	Trx-hSOD1-WT-his	E-hSOD1	apo-hSOD1	apo-hSOD1-monomer
19_c_	conf #2	1.3	0.2	1.7	6.1
41_c_	conf #1	5.6	6.5	5.4	3.0
120_c_	conf #1	2.0	1.0	2.6	8.9
22_c_	conf #4	4.1	1.1	5.0	none
56_c_	conf #3	5.2	6.8	4.1	none
155_c_	conf #5	7.3	2.2	1.6	none
16_L-40_	aa 40–47	5.6	none	none	none
3_L-42_	aa 42–49	6.7	none	none	none
37_L-63_	aa 63–71	2.1	none	6940	none
11_L-80_	aa 80–88	5.5	none	none	None
33_L-112_	aa 112–121	5.0	none	none	None

a For clarity in the manuscript, antibody number has a subscript to denote epitope: conformation-dependent epitope (C) and linear epitope (L and the first amino acid number of the epitope).

b epitope was narrowed to ten amino acids (aa) or less for linear epitopes with the aa numbers listed and conformation-dependent epitopes (conf) were categorized with competitive binding assays into different categories numbered 1 through 5 (see Figure 2).

To further understand the HuMab interactions with hSOD1, two additional forms of hSOD1 were tested that lacked a fusion protein and had zinc and copper removed. A variant of hSOD1 with the two free cysteines altered to preclude intramolecular disulfide interchange (C6A/C111S) was produced in bacteria without fusion proteins or epitope tags [31] and was demetallated to remove any associated metals (apo-hSOD1) [32]. A monomeric version was generated by engineering two point mutations (F50E/G51E) within the dimer interface of the variant with the two free cysteines removed which was also demetallated to remove any associated metals (apo-hSOD1-monomer). Avidity was determined for each HuMab for apo-hSOD1 and apo-hSOD1-monomer. None of the five HuMabs recognizing linear epitopes demonstrated significant binding to apo-hSOD1 or apo-hSOD1-monomer (Table 1). This suggested that removal of the metal or generation of monomer did not allow binding of linear-epitope HuMabs. There were two distinct patterns seen with conformation-dependent HuMabs. Three HuMabs, 22_c_, 56_c_, and 155_c_, bound apo-hSOD1 with similar avidity as E-hSOD1, but they failed to bind apo-hSOD1-monomer (Table 1) suggesting that these HuMabs may require an intact SOD1 dimer for binding. In contrast, HuMabs, 19_c_, 41_c_, and 120_c_ bound the apo-hSOD1-monomer with K_D_ similar to apo-hSOD1, E-hSOD1, and Trx-hSOD1-WT-his (Table 1). Similar results were obtained when binding was assessed with a capture ELISA (data not shown). When no binding or low binding (6.9 µM for 37_L-63_ and apo-hSOD1) was detected with the Octet biosensor, no binding was detected in the ELISA. Antibody and protein combinations with K_D_ from 0.2 to 9 nM as measured on the Octet demonstrated strong signals in ELISA (data not shown).

### Binding to WT and Mutant hSOD1 Produced in Mammalian Cells

**Figure 3:**
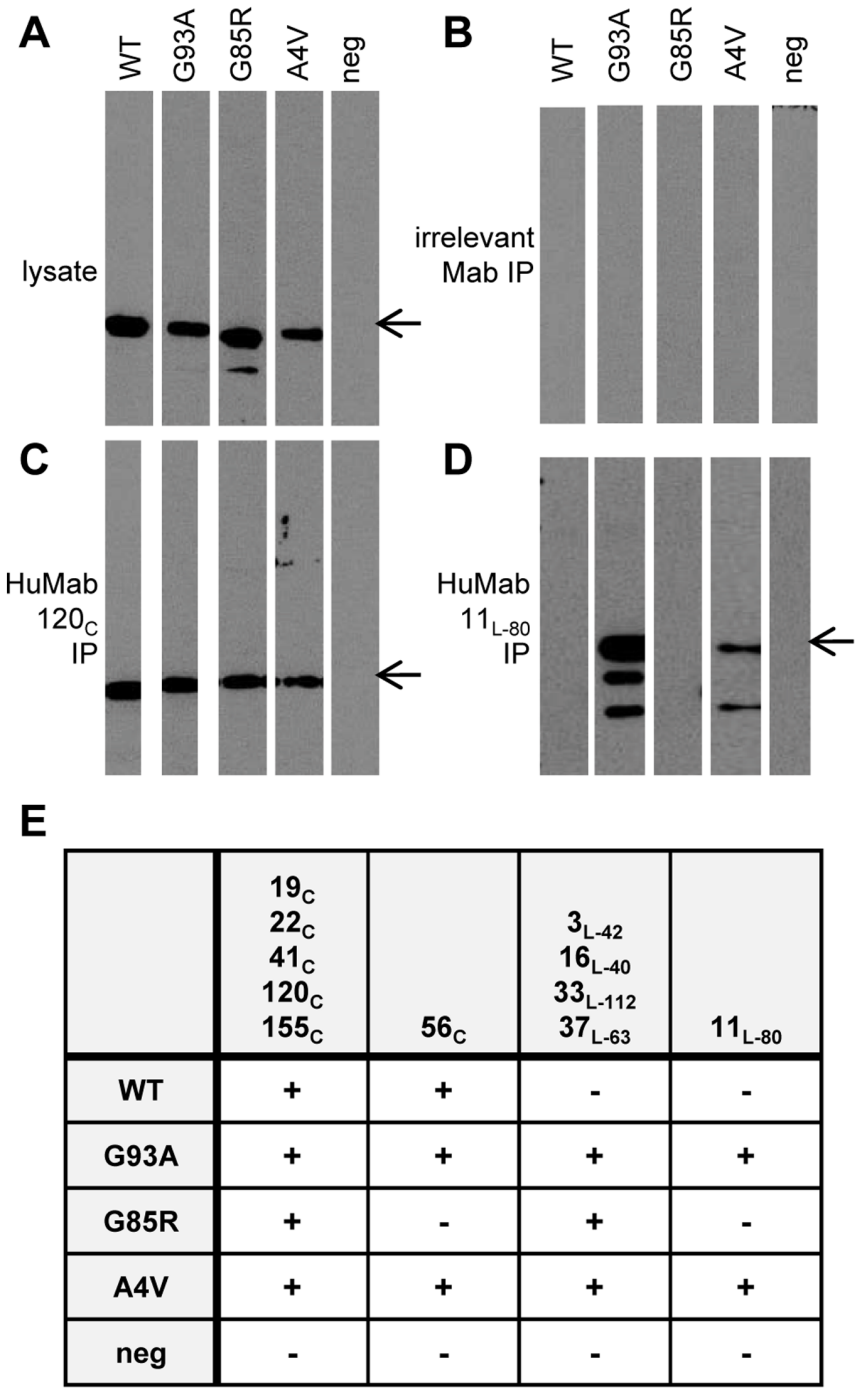
HuMab immunoprecipitation of WT and mutant hSOD1 from mammalian cells. A human derived cell line (293T) was transiently transfected with vectors engineered to express myc-tagged wild type (WT) or mutant hSOD1 (A4V, G85R, or G93A), or with empty vector as a control (neg). (A) Lysate from transfected cells was subjected to SDS-PAGE and immunoblot. Myc tagged proteins were detected with a mouse monoclonal antibody specific for the myc tag followed by goat anti-mouse HRP conjugate and chemiluminescence. An arrow to the right of the blots indicates a band present at the expected size for SOD1 (16 kDa). Lysate from transfected cells was mixed with an irrelevant isotype-matched antibody (B), HuMab 120_c_ (C), and HuMab 11_L-80_ (D) and incubated at ambient temperature for 2 hrs followed by immunoprecipitation (IP) with protein A sepharose beads. Precipitated material was subjected to SDS-PAGE and immunoblotted with the anti-myc antibody. (E) Additional immunoprecipitations where performed with the remaining HuMabs, and the presence or absence of a band at the appropriate size for SOD1 in anti-myc immunoblots is indicated with a plus or minus.

To assess HuMab recognition of WT and mutant proteins produced in a mammalian system, we used transient transfection of human 293T cells to produce WT and mutant (A4V, G85R and G93A) hSOD1 tagged with a C-terminal myc tag. Lysate was generated from transfected cells and HuMab binding assessed using immunoprecipitation, followed by immunoblot detecting the myc tag. The backbone plasmid (pcDNA) was transfected as a negative control, and each of the lysates was incubated with an irrelevant HuMab followed by immunoprecipitation as an additional negative control. When lysate was directly subjected to immunoblot and probed with an anti-myc antibody, a band was detected at the appropriate size for hSOD1 from the transfected WT, A4V, G85R, and G93A plasmids, but not for the negative-control plasmid (Figure 3A) confirming that each protein was expressed at similar levels. No myc tagged hSOD1 protein was detected when the lysates were immunoprecipitated with an irrelevant antibody (Figure 3B). The conformation-dependent HuMab, 120_c_, immunoprecipitated all hSOD1-myc proteins tested (Figure 3C). All conformation-dependent HuMabs with the exception of 56_c_ were able to bind all hSOD1-myc proteins tested (summarized in Figure 3E). hSOD1-G85R-myc was not immunoprecipitated by 56_c_, and the lack of 56_c_ recognition of the G85R mutant hSOD1 was confirmed by ELISA with GST-hSOD1-G85R (data not shown). The linear HuMab, 11_L-80_, did not immunoprecipitate the hSOD1-myc or the hSOD1-G85R-myc but did immunoprecipitate hSOD1-A4V-myc and hSOD1-G93A-myc (Figure 3D). The lack of 11_L-80_ recognition of WT hSOD1 agreed with the lack of 11_L-80_ binding to E-hSOD1 (Table 1). GST-hSOD1-G85R was used in an ELISA assay to confirm the lack of 11_L-80_ binding (data not shown). The G85R mutation falls in the center of the identified epitope for 11_L-80_, amino acids 80 to 88. Because HuMab 11_L-80_ recognizes a linear epitope, it is able to recognize any fragments of the hSOD1 protein generated during the cell lysis procedure which are found as lower bands in the immunoblot. The other linear HuMabs bound all the mutant hSOD1-myc proteins but not the WT (summarized in Figure 3E) in agreement with the lack of binding to E-hSOD1 (Table 1). The binding of the linear-epitope HuMabs to hSOD1 point mutants expressed in the mammalian cells suggests that mutant hSOD1 adopts a conformation similar to the Trx-hSOD1-WT-his protein which is different than that of WT hSOD1 produced in mammalian cells. A possible explanation is that some proportion of both hSOD1 mutants expressed in mammalian cells and Trx-hSOD1-WT-his expressed in *E. coli* is misfolded or unfolded. Previous data has determined that both A4V and G93A hSOD1 are substantially destabilized with G93A fully unfolded at 37°C [35]. Our linear-epitope HuMabs did not bind hSOD1-WT-myc expressed in our mammalian cell system suggesting that there was not enough misfolded or unfolded protein for detection in our transient expression system. This contrasts with previous publications documenting misfolding of WT hSOD1 protein with transient expression [36] suggesting that differences in level of hSOD1 expression or different epitope tags or fusion proteins could affect amounts of WT misfolded protein.

### hSOD1 Hydrophobic Exposure and HuMab Inhibition

**Figure 4:**
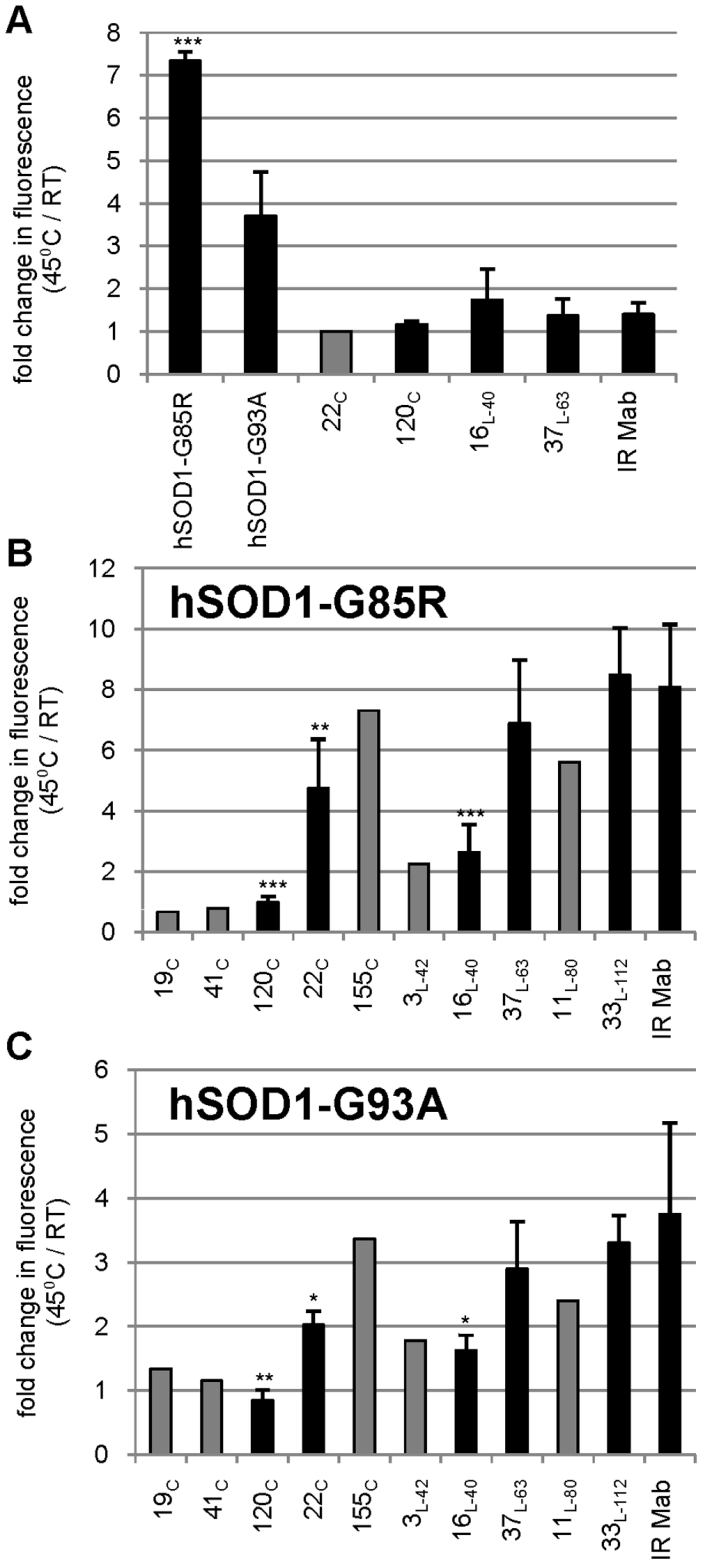
Increased hydrophobicity of hSOD1 can be inhibited by a subset of hSOD1 specific HuMabs. (A) hSOD1-G85R, hSOD1-G93A, various hSOD1-specific HuMabs, and an irrelevant isotype-matched antibody (IR Mab) were separately incubated with 5 mM EDTA at room temperature (RT) or 45°C for 4 hrs. Hydrophobicity was measured with ANS dye fluorescence using a Victor 3 plate reader at 390 nm excitation and 460 nm emission and is reported as the fold change in relative fluorescence units from RT to 45°C. hSOD1-G85R (B) and hSOD1-G93A (C) were mixed with irrelevant antibody or various HuMabs with 5 mM EDTA and incubated at RT or 45°C for 4 hrs. Hydrophobicity was measured as indicated above. The mean of replicates is noted with a thick black bar with the standard deviation indicated. Samples with insufficient material for replicates are indicated with the single data point as a thick grey bar. Statistical significant differences from irrelevant antibody were determined using a Student’s t-test with P<0.01 (***), <0.05 (**), and <0.10 (*) indicated above the black bar for each sample.

Mutations in hSOD1 induce a propensity to misfold under certain conditions and lower the thermodynamic barrier to misfolding or unfolding [35], [37]. It is possible that antibody binding could stabilize hSOD1 and reduce the propensity toward misfolding, unfolding, and aggregation. Or, antibody binding could stabilize misfolded or unfolded hSOD1 and increase aggregation. To assess misfolding, unfolding and aggregation, we measured changes in hydrophobicity of mutant hSOD1 with exposure to increased temperature and EDTA using the dye ANS which yields increased fluorescence with increased hydrophobicity. Protease cleavage was used to remove the GST tag from GST-hSOD1-G85R and GST-hSOD1-G93A. Protease, uncleaved GST fusion protein, and GST tag were removed with glutathione beads to yield purified hSOD1-G85R and hSOD1-G93A. Mutant hSOD1 proteins, hSOD1-specific HuMabs, and an irrelevant isotype-matched antibody were separately incubated with 5 mM EDTA at room temperature or at 45°C for 4 hrs. The proteins were cooled to room temperature, mixed with ANS, and analyzed with a fluorescent plate reader. hSOD1-G85R and hSOD1-G93A both produced a fluorescent signal increase of approximately 7-fold and 4-fold (respectively) when heated at 45°C as compared to room temperature (Figure 4A). In contrast, the HuMabs tested displayed no significant change in fluorescent signal from room temperature to 45°C (Figure 4A and data not shown). To assay the effect of HuMabs on the hydrophobic exposure of mutant hSOD1, we mixed hSOD1-G85R with various HuMabs in the presence of 5 mM EDTA and incubated for 4 hrs at room temperature or 45°C followed by analysis of hydrophobicity with ANS. Irrelevant antibody mixed with hSOD1-G85R had an 8-fold increase in fluorescence upon heating (Figure 4B) similar to that seen with hSOD1-G85R alone (Figure 4A). A similar large increase in fluorescence was obtained when hSOD1-G85R was separately mixed with HuMabs 155_c_, 37_L-63_, or 33_L-112_ and heated to 45°C. In contrast, HuMabs 19_c_, 41_c_, and 120_c_ separately mixed with hSOD1-G85R gave no increase in fluorescence when heated to 45°C for 4 hr (Figure 4B). An intermediate result was obtained with 16_L-40_ and 3_L-42_ mixed with hSOD1-G85R: fluorescence increased when heated, but only 2.5-fold rather than 8-fold (Figure 4B). The fluorescence increase of 22_c_ and 11_L-80_ also gave an intermediate result, but with a lower statistical difference from irrelevant antibody (P<0.05) (Figure 4B). Although the magnitude of fluorescent change from RT to 45°C was not as great with hSOD1-G93A as it was with hSOD1-G85R (3.5-fold versus 8-fold), a similar pattern was seen for all HuMabs when mixed with hSOD1-G93A and heated to 45°C (Figure 4C). Irrelevant antibody, 155_c_, 37_L-63_, and 33_L-112_ all had a 3-fold or greater increase in fluorescence with heating to 45°C while HuMabs 19_c_, 41_c_, and 120_c_ gave no increase. The intermediate increases of 16_L-40_, 3_L-42_, 22_c_ and 11_L-80_ separately mixed with hSOD1-G93A had a lower statistical difference from irrelevant antibody (P<0.10) (Figure 4C). When mutant hSOD1 was heated prior to addition of HuMabs 19_c_, 41_c_, and 120_c_, there was a significant fluorescence increase similar to the irrelevant antibody with mutant hSOD1 (data not shown). This suggests that antibody binding is required prior to exposure of SOD1 to denaturing conditions to prevent hydrophobic exposure. In summary, the three conformation-dependent HuMabs that could bind apo-hSOD1-monomer (Table 1) were able to prevent hydrophobic exposure upon heating possibly through stabilization of monomer. The two linear-epitope HuMabs with overlapping epitopes, 16_L-40_ and 3_L-42_, reduced hydrophobic exposure which could be reduction in aggregation due to the binding of hSOD1 beta-sheet four. The linear-epitope HuMab 11_L-80_ and conformation-dependent HuMab 22_c_ may provide a reduction in hydrophobic exposure although the results were less significant.

### Antibody Delivery to hSOD1-G93A Transgenic Mice

**Figure 5:**
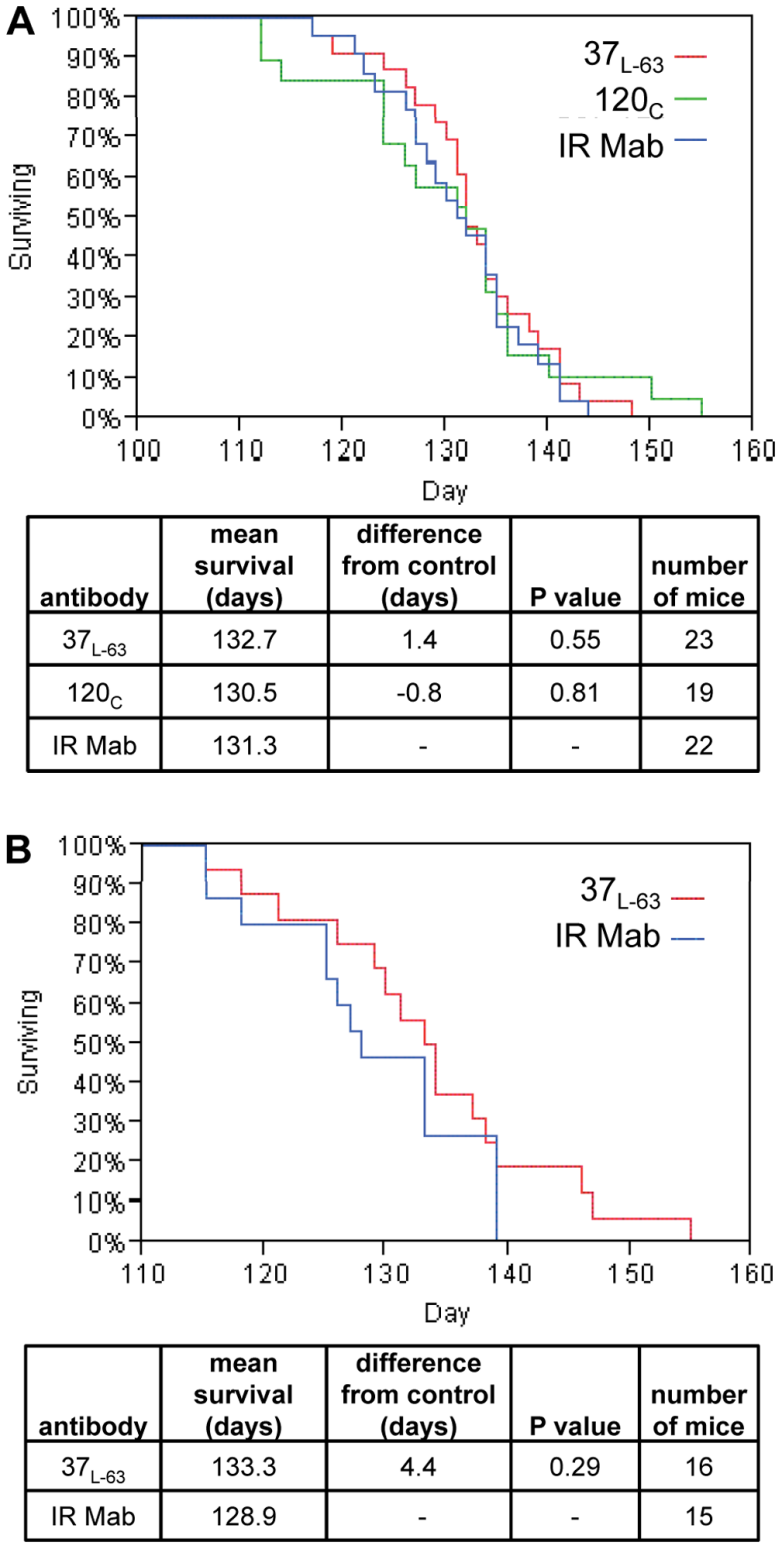
Antibody delivery to hSOD1-G93A transgenic mice. (A) HuMabs 37_L-63_ (red) and 120_c_ (green) and an irrelevant isotype-matched HuMab (IR Mab, blue) were delivered to the lumbar intrathecal space of hSOD1-G93A transgenic mice via osmotic pump from mouse age 65 to 115 (days). Complete two limb paralysis was used as an endpoint of disease and the day of disease endpoint was used to calculate the percent survival each day. For each group, the mean days to reach the endpoint (mean survival) and P value from the Mantel-Cox test (log-rank) were calculated using JMP and are listed below the graph. (B) HuMab 37_L-63_ (red) and an irrelevant isotype-matched HuMab (IR Mab, blue) were delivered by intraperitoneal injection to hSOD1-G93A transgenic mice. Antibody dosing was initiated at mouse age of 65 days and continued once per week for the duration of the mouse survival. Endpoint and statistics were calculated as above.

A commonly used transgenic hSOD1 mouse model with a high-copy number of hSOD1-G93A (B6SJL-Tg(SOD1G93A)1Gur/J strain) [17] was used to determine the therapeutic potential of anti-hSOD1 HuMabs. A small exploratory experiment with two to five mice per HuMab was performed to assess potential adverse effects of HuMab delivery into mouse cerebrospinal fluid. The hSOD1-specific HuMabs and an irrelevant HuMab (ten mice) were delivered intrathecally by surgical implantation of an osmotic pump with a lumbar intrathecal (IT) catheter as performed previously for RNAi delivery in this model system [34]. Antibody delivery was initiated at day 65 (mouse age) prior to onset of symptoms and continued until day 115 when the pump mechanism was exhausted and it was removed. Animals were observed daily for the endpoint criteria of complete two limb paralysis. Antibody delivery for 50 days was completed for all tested HuMabs with no obvious toxicity (data not shown). The animal group size in this experiment was too small to yield statistical significance in delay of disease endpoint versus an irrelevant antibody (Table S4 in File S1). Therefore, two HuMabs with the longest delay in disease endpoint in the pilot experiment were further tested with larger animal groups. HuMab 120_c_, HuMab 37_L-63_, and an irrelevant antibody were each delivered IT to 19 to 23 mice per antibody. The duration to disease endpoint from birth was 131 days for irrelevant antibody and 131 and 133 days for HuMabs 120_c_ and 37_L-63_ respectively (Figure 5A). The change from irrelevant antibody was not statistically significant for either HuMab120_c_ or 37_L-63_. For the small pilot experiment (Table S4 in File S1), the average duration to disease endpoint from birth was 133 days for both HuMabs 120_c_ and 37_L-63_. The results are similar to those obtained with the pilot experiment, however, the average duration to disease endpoint for the irrelevant antibody was 122 days for the pilot experiment (n  = 10, Table S4 in File S1) versus 131 days for the larger experiment (n  = 22, Figure 5B). This difference could be due to sampling error with a smaller group or because care was not taken to distribute siblings amongst different groups in the pilot experiment which was corrected in the larger group experiment. Residual antibody remaining in the osmotic pump at time of removal from the 50 day dosing in a mouse was tested in ELISA for binding to hSOD1 and the activity was found to be equivalent to antibody stored at 4°C for the same duration of time (Table S5 in File S1).

An alternate delivery method of weekly intraperitoneal (IP) injection was also tested. IP injection of 50 mg/kg of HuMab 37_L-63_ and an irrelevant antibody was initiated at day 65 and continued weekly throughout the experiment to groups of 16 or 15 hSOD1-G93A mice respectively. The mean duration to disease endpoint was 129 days for irrelevant antibody and 133 days for HuMab 37_L-63_ (Figure 5B). The four-day extension of mean disease endpoint for the HuMab 37_L-63_ treated group did not reach statistical significance.

To assess HuMab distribution and concentration in these two experimental models, we retrospectively analyzed available sera and spinal cord tissue that had been collected from mice at the time of disease endpoint. A quantitative anti-human antibody ELISA was performed to measure levels of HuMabs in the serum and tissue. Spinal cord lysate was analyzed for all mice that received IT delivery and was expressed as ng of HuMab per mg of total protein in the lysate. For the majority of the mice (tissue sample harvested from 2 to 34 days post-pump removal), the concentration of HuMab was below the limit of detection (<1.5 ng/mg) (Table S6 in File S1). Only two mice had levels of HuMab >1.5 ng/mg after pump removal with 46.6 ng/mg present one day after pump removal and 1.8 ng/mg present at 6 days after pump removal. Three of the mice had tissue samples harvested prior to pump removal, and all three had detectable levels of HuMab from 1.4 to 11.8 ng/mg. HuMab level in serum was tested for six mice. Two mice were tested at 20 days after pump removal (end of antibody dosing) and had 0.3 µg/ml and 81 µg/ml of HuMab detectable in serum (Table S6 in File S1). The other four assayed mice ranged from 23 to 34 days after pump removal and had HuMab concentration in the sera below the limit of detection (<0.15 µg/ml). It is possible that the mouse with 81 µg/ml of HuMab in the serum at 20 days after pump removal had a misplaced catheter that delivered the HuMab systemically.

For IP delivery, spinal cord lysate had 41 and 20 ng of HuMab per mg of total protein for two mice treated with irrelevant antibody and 2 and 5 ng/mg for two mice treated with HuMab 37_L-63_ (Table S7 in File S1). For IP delivery, HuMab serum level was analyzed in three mice and ranged from 200 to 800 µg/ml. We limited the sera collection during the experiment because of concerns that it would affect the disease course so a separate experiment was done to verify consistent levels of antibody in sera from IP dosing. Isotype-matched irrelevant HuMab was dosed IP at 50 mg/kg once a week in normal mice over a course of 10 weeks, and gave a similar range of a serum HuMab levels throughout the entire time of dosing (Table S8 in File S1). These mice did develop anti-human antibodies after the fourth week of injections, but this did not lower the average HuMab serum concentrations at day 7 after injection through the length of the experiment.

To provide a more direct comparison of delivery methods to different regions of the spinal cord and brain, irrelevant human antibody was delivered to normal mice IT (n  = 5) and IP (n  = 3) for two weeks and human antibody level was quantified from sera and different regions of the spinal cord and brain. Mice were perfused with PBS prior to the tissue harvest for both delivery methods. With IP injection, high levels of human antibody were detected in the sera (544±47 ug/ml, average of three mice) and antibody was evenly distributed in the lumbar and cervical spinal cord and four different regions of brain tissue at 26 to 39 ng/mg total protein (Table S9 in File S1). With IT pump delivery, antibody was present primarily in lumbar spinal cord (13±5 ng/mg, average of five mice) with a lower level in cervical spinal cord (2±2 ng/mg) and below the limit of detection in the four different sections of brain (<1.5 ng/mg). Antibody was present at an average of 51±4 µg/ml in the sera of the five IT-treated mice. From these measurements, we conclude that the IP delivery method gave higher levels of antibody in the nervous tissue and could be delivered for a longer period of time in the disease model with fewer complications from surgery. However, we were not able to completely rule out that the levels of antibody detected in the spinal and brain tissue is an artifact from the high levels in the blood with subsequent contamination of the tissues during dissection and removal.

## Discussion

**Figure 6:**
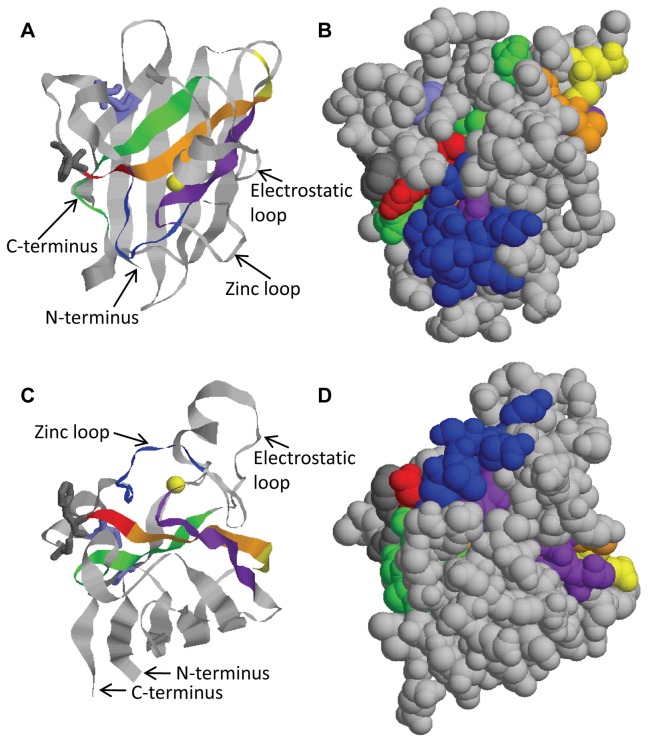
HuMab epitope location in predicted hSOD1 structure. (A) The crystal structure of one monomer of a hSOD1 dimer (2C9V) is indicated in ribbon model from Rasmol. The bound zinc is indicated as a yellow sphere. The two cysteines involved in the intramolecular disulfide bond (C57 and C146) are displayed as wireframe in light blue. The phenylalanine at position 50 and glycine at position 51, two residues mutated at the dimer interface to generate apo-hSOD1-monomer, are displayed as wireframe in dark grey. HuMab epitopes are indicated as follows: 16_L-40_ amino acids 40–47 in yellow, 3_L-42_ amino acids 42–49 in red, the overlap of these two epitopes is orange, 37_L-63_ amino acids 63–71 in dark blue, 11_L-80_ amino acids 80–88 in purple, and 33_L-112_ amino acids 112–121 in green. (B) The same orientation molecule in (A) is displayed as a space-filling model. (C) The ribbon model from (A) is rotated 90 degrees on the x-axis to view from the bottom of the molecule in (A). (D) The same orientation molecule in (C) is displayed as a space-filling model.

We have selected hSOD1 specific HuMabs with a range of distinct epitopes. The five HuMabs recognizing linear epitopes were only able to bind some form of misfolded or unfolded hSOD1. The lack of binding to properly-folded hSOD1 agrees with the location of the epitopes in the three-dimensional structure of hSOD1 (Figure 6) [11]. Four of the epitopes (16_L-40_, 3_L-42_, 11_L-80_, and 33_L-112_) are located within the beta-sheets that form the back of the metal-binding pocket. HuMabs 16_L-40_ and 3_L-42_ bind in a region (amino acids 42–48) that was previously characterized with a polyclonal antibody (USOD) generated to the peptide. Similar to what we have found with the monoclonal antibodies, 16_L-40_ and 3_L-42_, USOD was not able to bind properly folded dimer or monomer hSOD1 but recognized unfolded hSOD1 [38]. USOD specifically stained inclusions within motor neurons in spinal cord tissue from two different SOD1 mutant fALS cases but not tissue from five different sALS cases [38]. We would anticipate that 16_L-40_ and 3_L-42_ would have similar binding, but this remains to be tested. The fifth epitope (37_L-63_) is present on one side of the zinc-binding loop and the other side of the same loop has a portion of the 11_L-80_ epitope. Very little of these epitopes would be accessible to antibodies as imaged with a space-filling model of the hSOD1 crystal structures (Figure 6 B and D). HuMab 37_L-80_ has the largest amount of the epitope visible in the space-filling model (Figure 6B), and is the only linear-epitope HuMab with measurable low avidity to apo-hSOD1 (Table 1). Each of the linear epitopes contains at least one amino acid involved in zinc or copper chelation. If the zinc-binding loop and the electrostatic loop that form the front of the metal-binding pocket were flexible (from loss of bound metals and/or disulfide bonding), the epitopes may become accessible as the loops move and allow access to the beta-sheets at the back of the metal-binding pocket. Previous studies exploring the crystal structure of A4V and G93A apo-hSOD1 [39] or the solution NMR structure of various forms of hSOD1 [40] suggest that there is large flexibility in the zinc-binding and electrostatic loops when metal is not bound. Interestingly, we found that the linear-epitope HuMabs could not bind apo-hSOD1. These same linear-epitope HuMabs did bind Trx-hSOD1-WT-his and GST-hSOD1-WT fusion proteins suggesting that the lack of metal may not provide enough structural disturbance for the binding of a large antibody molecule and a second structural change such as a point mutation like A4V or G93A or attachment of a fusion protein is required for HuMab binding. Alternatively, the intramolecular disulfide bond may need to be reduced in combination with lack of metal to produce enough flexibility for antibody binding. Future structural studies with hSOD1 bound by linear-epitope HuMabs could provide understanding of the exact hSOD1 conformation required for HuMab binding.

The identified hSOD1 specific HuMabs had two distinct groups of conformation-dependent epitopes that presumably bind non-linear epitopes. One group of HuMabs (22_c_, 56_c_, and 155_c_) had an epitope that was only available on hSOD1 dimer. These antibodies either bound an epitope that spans the dimer interface, or that has a conformation-dependent epitope only present on the dimer. The other group of conformation-dependent HuMabs (19_c_, 41_c_, and 120_c_) had an epitope that was available on hSOD1 monomer and dimer. Only the three HuMabs that could bind to hSOD1 monomer were able to prevent any increase in hydrophobic exposure from mutant hSOD1 subjected to denaturing conditions. The epitope bound by these three HuMabs could be a key region on hSOD1 for stabilizing the properly-folded protein and thus preventing unfolding. Or, binding to monomer could be a key step in preventing hSOD1 misfolding and aggregation as monomerization is likely an early step in the misfolding pathway *in vitro* [41]. A polyclonal antibody specific for the exposed dimer interface (SEDI) is able to recognize hSOD1 in inclusion structures within neurons suggesting that monomeric hSOD1 is present in ALS patient tissue [42], [43]. It is not clear if binding and stabilizing monomer would translate directly to a benefit *in vivo* as other aspects may be important for disease treatment. However, a recent report of a 40 day increase in survival time in G37R hSOD1 transgenic mice with SEDI peptide immunization is suggestive that monomer recognition may have a benefit *in vivo* [25].

The binding of both the linear and conformation-dependent HuMabs to Trx-hSOD1-WT-his purified from bacteria suggests that the protein has enough intact structure for the conformation-dependent HuMab binding and is flexible enough to expose the linear epitopes at the back of the metal-binding pocket. The concept that the protein has to change from the native conformation to allow binding of the linear-epitope HuMabs is supported by lack of all conformation-dependent HuMab binding to Trx-hSOD1-WT-his if any of the linear-epitope HuMabs are pre-bound to hSOD1. This ability to lock the protein in a conformation that is not recognized by the other group of HuMabs is reciprocal. This panel of HuMabs should prove useful to probe the structure of hSOD1 in solution as hSOD1 may only expose some conformations transiently which could be trapped by an antibody for further study. Similarly, hSOD1 conformations could be assessed in complex samples such as patient tissue, sera or spinal fluid where there may be multiple forms and versions of hSOD1 protein [44].

Unfortunately, due to the prohibitive high cost of the *in vivo* experiments, we were not able to test the entire panel of eleven HuMabs in an ALS animal model. We selected one linear-epitope HuMab, 37_L-63_, and a conformation-dependent HuMab, 120_c_, that bound to hSOD1 dimer and monomer. The G93A high-copy mouse model was used because of the rapid disease onset and progression with an average survival of ∼130 days. This allows for a shorter period of antibody treatment that fits within the limits of the osmotic pump delivery time. The rapid onset of symptoms is likely due to the higher level of mutant hSOD1 expression because of the higher transgene copy number. This yields a level of mutant hSOD1 that is approximately 20-fold higher than in human ALS and thus may pose a greater challenge for treatment [17]. Our *in vivo* testing results did not replicate the previous data with passive immunotherapy of mutant hSOD1 transgenic mice via osmotic pump, which provided a 5 to 9 day survival benefit [23], [26]. However, there are many different variables that may contribute to the lack of benefit in our experiment: route of administration (IT versus intraventricular), disease endpoint (complete two limb paralysis versus 30 seconds to right), genetic background of the mouse (B6SJL versus C57BL/6), species of the antibody (human versus mouse), and epitope of the antibody. In our testing we chose IT delivery because this delivery of siRNA had afforded distribution throughout the mouse central nervous system (CNS) and demonstrated a statistically significant survival extension [34]. However, with IT delivery, we found that antibody was present in the lower spinal cord and was cleared very quickly from the spinal cord after pump removal. Previous passive immunotherapy in ALS mouse models delivered antibody into the left ventricle of the brain [23], [26]. It is possible that this location of delivery and distribution is required in the G93A hSOD1 transgenic mouse model to demonstrate an extension in survival. It is not clear if distribution of the antibody throughout the spinal cord and brain would be required in treatment of the human disease as the levels of mutant hSOD1 are much lower than the G93A hSOD1 transgenic mouse model.

To address some of the issues with the IT antibody delivery and distribution, we tested an alternative method of delivery by IP injection, which allowed the antibody to be dosed to the disease endpoint even when the mice would have been too weak to carry the additional weight of an osmotic pump. The IP delivery of HuMab 37_L-63_, gave an encouraging trend toward extended survival that was not statistically significant. We found detectable, evenly distributed HuMab in the mouse CNS suggesting that this route may provide antibody delivery to the brain without invasive surgery. HuMab 37_L-63_ levels in the serum and spinal cord were five- to ten-fold lower than an irrelevant isotype-matched antibody (Table S7 in File S1) suggesting that this specific antibody may have a solubility, transport, or half-life issue that lower the available antibody for therapeutic efficacy. Given the encouraging trend seen with HuMab 37_L-63_, IP delivery of other HuMabs such as one with the capacity to bind to apo-hSOD1-monomer is an attractive next step to provide a clearer demonstration of *in vivo* efficacy. HuMab IP delivery to ALS mouse models with longer time for disease onset (G37R, G85R, or low-copy G93A) and endpoints other than survival extension (reduction in motor neuron loss, reduction in mutant hSOD1, or muscle strength and motor assessments) may afford a clearer picture of HuMab efficacy. This was recently demonstrated with immunization of the SEDI peptide not providing a significant change in duration of survival for high-copy G93A hSOD1 transgenic mice while a statistically significant extension of 40 days of survival was seen with G37R hSOD1 transgenic mice [25]. Bapineuzumab and solanezumab are both in human clinical trials and are dosed systemically to patients for Alzheimer’s disease treatment suggesting that systemic HuMab delivery could be a viable method for antibody treatment of other CNS disease targets [45].

It has proven extremely difficult to translate treatments from ALS animal models into humans [20]. A prime example is Riluzole, the only approved drug for ALS, providing variable results in the G93A transgenic mouse model with a modest extension of 14 days for survival [21] to no statistically significant extension of survival [22]. It may be that the animal models are not able to recapitulate all of the factors involved in the human pathology and disease or that there are differences in treating SOD1 mutant ALS and sALS. This presents a challenge when selecting compounds to introduce into human clinical trials. Recent publications detecting higher levels of a subset of SOD1 antibodies in long-term survivor ALS patients [46], misfolded SOD1 in sALS [7], [9], and SOD1 misfolding when FUS and TDP43 are mislocalized [47] provide evidence that SOD1 has potential as a therapeutic target in fALS and sALS.

## Supporting Information

File S1 **Tables S1, S2, S3, S4, S5, S6, S7, S8, S9.**
(DOC)
