# Supplementary material for: Identification of Human Monoclonal Antibodies Specific for Human SOD1 Recognizing Distinct Epitopes and Forms of SOD1
Source: PLoS One. 2013 Apr 17;8(4):e61210. doi: 10.1371/journal.pone.0061210 (PMC3629177; doi:10.1371/journal.pone.0061210)
Supplement: File S1 — Tables S1, S2, S3, S4, S5, S6, S7, S8, S9. (DOC) [file pone.0061210.s001.doc]

**Table S**1. Mouse immunizations to generate SOD1 HuMabs.

| **antibody** | **mouse number** | **first immunogen** | **# of immunizations** | **second immunogen** | **# of immunizations** |
| --- | --- | --- | --- | --- | --- |
| **41** | 189114 | GST-hSOD1-A4V^a^ | 9 | - | - |
| **155** | 176306 | E-hSOD1^b^ | 14 | - | - |
| **3** | 176311 | E-hSOD1 reduced^c^ | 12 | GST-hSOD1-WT | 8 |
| **19** | 176312 | E-hSOD1 reduced | 12 | GST-hSOD1-WT | 8 |
| **56** | 176312 | E-hSOD1 reduced | 12 | GST-hSOD1-WT | 8 |
| **11** | 180358 | GST-hSOD1-WT | 13 | - | - |
| **22** | 180358 | GST-hSOD1-WT | 13 | - | - |
| **33** | 181591 | Trx-hSOD1-A4V-his^d^ | 14 | - | - |
| **37** | 181591 | Trx-hSOD1-A4V-his | 14 | - | - |
| **16** | 181595 | GST-hSOD1-WT | 7 | - | - |
| **120** | 181597 | GST-hSOD1-WT | 7 | - | - |

^a^ Amino-terminal glutathione sulfur transferase (GST) fusion to human SOD1 (hSOD1) with the alanine at position four changed to valine (A4V).

^b^ hSOD1 purified from erythrocytes (E-hSOD1).

^c^ E-hSOD1 was reduced with dithiothreitol and free cysteines were capped with N-ethylmaleimide. Size exclusion chromatography was used to confirm that >50% of the protein was monomeric.

^d^ Amino-terminal thioredoxin (TRX) fused to hSOD1 with an A4V mutation and a carboxy-terminal six histidine tag.

**Table S2. HuMab heavy chain variable region sequences.**

| **Antibody** | **V_H_** | **CDR1** | **CDR2** | **CDR3** |
| --- | --- | --- | --- | --- |
| **41** | 1-18 | SFGIS | WISVYNDYTNYAQKFQG | KRGGDMDY |
| **155** | 5-51 | SYWIG | IIYPGDSDTRYSPSFQG | QGSGWYGNYFDY |
| **3** | 4-34 | GYYWN | EIHQSGGPHYNPSLKS | LDDY |
| **19** | 1-69 | NFVIG | RIIPILDIANYAQKFQG | TGNYYKPYDY |
| **56** | 1-69 | SYAIS | RIIPILGTAKYAQKFQG | DQDYYGMDV |
| **11** | 3-33 | NYGIH | IIWHDGSNSYYVDSVKG | IIGGAFDI |
| **22** | 3-33 | SYGMH | LIWYDGSNKYYADSVKG | EGFNWDAFDI |
| **33** | 3-07 | RYWMS | NIKQDGSETHYVDSVKG | GDY |
| **37** | DP-44 | RYALH | AIGIGGGTFYADSVKG | DTYYDFFDAFDI |
| **16** | DP-44 | SYSMH | AIGTAGGTYYADSVKG | EYFFGSGNYGY |
| **120** | 3-07 | GYWMS | NIKQDGGEKYYGDSVKG | AGGLDY |

**Table S3.** **HuMab light chain variable region sequences.**

| **Antibody** | **V_κ_** | **CDR1** | **CDR2** | **CDR3** |
| --- | --- | --- | --- | --- |
| **41** | L5 | RASQDISSWLA | LASSLQS | QQANSFPWT |
| **155** | A27 | RASQSFSRGYLA | GASSRVT | QQYDSSPYT |
| **3** | L6 | RASQSVSSYLA | NASNRAT | QQRSNWPRT |
| **19** | A26 | RASQSIGSSLH | YASQSFS | HQSSSLPIT |
| **56** | L5 | RASQGISSWLA | AASSLQS | QQTNNFPWT |
| **11** | L15 | RASQGISSWLA | AASSLQS | QQYNSYPIT |
| **22** | A27 | RASQSVRISYLA | GTFSRAT | QQYGSSMYT |
| **33** | L15 | RASQDISSWLA | AASSLQS | QQYKSYPLT |
| **37** | L15 | RASQGISSWLA | AASSLQS | QQYDSYPLT |
| **16** | L6 | RASQSVSSYLA | DASNRAT | QQRSNWPPT |
| **120** | L6 | RASQSVSSYLA | DASNRAT | QQRSNWYT |

**Table S**4. Small group G93A hSOD1 mouse experiments.

|  | **mean survival (days)** | **difference from control (days)** | **number of mice** | **P value** |
| --- | --- | --- | --- | --- |
| **155_C_** | 117.5 | -4.3 | 2 | 0.76 |
| **41_C_** | 121.3 | -0.5 | 3 | 0.47 |
| **3_L-42_** | 124.5 | 2.7 | 4 | 0.84 |
| **11_L-80_** | 126.3 | 4.5 | 4 | 0.10 |
| **22_C_** | 127.6 | 5.8 | 4 | 0.37 |
| **33_L-112_** | 129.3 | 7.5 | 4 | 0.72 |
| **19_C_** | 129.3 | 7.5 | 4 | 0.12 |
| **16_L-40_** | 131.0 | 9.2 | 4 | 0.08 |
| **120_C_** | 133.2 | 11.4 | 5 | 0.11 |
| **37_L-63_** | 133.3 | 11.5 | 4 | 0.10 |
| **IR Mab** | 121.8 | - | 10 | - |

**Table S5. Antigen binding activity of pump residual antibody.**

|  | **storage** | **Mean mg/ml - OD280** | **Mean mg/ml - ELISA^a^** |
| --- | --- | --- | --- |
| **120_C_** | 4 degrees C | 12.7 | 12.7 |
| **120_C_** | pump residual | 21.3 | 19.8 |
| **37_L-63_** | 4 degrees C | 9.2 | 9.2 |
| **37_L-63_** | pump residual | 20 | 18.5 |
| **IR Mab** | 4 degrees C | 10.8 | 10.8 |
| **IR Mab** | pump residual | 16.2 | 15.6 |

^a^ Residual antibody remaining in the pump after removal from the mouse (50 day) for four mice per each antibody was assayed by quantitative antigen binding ELISA with antibody stored at 4 degrees Celsius as the standard curve.

**Table S6. HuMab concentration in spinal cord and sera of IT dosed hSOD1-G93A mice.**

| **mAb** | **age (days) at disease endpoint** | **age (days) pump removed** | **days from pump removal to collection of spinal tissue** | **ng of HuMab per mg of total protein in spinal cord lysate** | **µg/ml HuMab in sera** |
| --- | --- | --- | --- | --- | --- |
| IR | 117 | 116 | 1 | 46.6 | nd^a^ |
|  | 121 | 115 | 6 | 1.8 | nd |
|  | 122 | 115 | 7 | <1.5 | nd |
|  | 123 | 115 | 8 | <1.5 | nd |
|  | 126 | 113 | 13 | <1.5 | nd |
|  | 127 | 115 | 12 | <1.5 | nd |
|  | 127 | 115 | 12 | <1.5 | nd |
|  | 128 | 116 | 12 | <1.5 | nd |
|  | 129 | 115 | 14 | <1.5 | nd |
|  | 130 | 116 | 14 | <1.5 | nd |
|  | 131 | 116 | 15 | <1.5 | nd |
|  | 132 | 116 | 16 | <1.5 | nd |
|  | 134 | 114 | 20 | <1.5 | nd |
|  | 134 | 114 | 20 | <1.5 | 0.3 |
|  | 135 | 115 | 20 | <1.5 | nd |
|  | 135 | 115 | 20 | <1.5 | nd |
|  | 135 | 115 | 20 | <1.5 | 81 |
|  | 137 | 113 | 24 | <1.5 | nd |
|  | 139 | 113 | 26 | <1.5 | nd |
|  | 141 | 116 | 25 | <1.5 | <0.15 |
|  | 141 | 114 | 27 | <1.5 | nd |
|  | 144 | 115 | 29 | <1.5 | nd |
| 120_C_ | 112 | 112 | 0 | 1.4 | nd |
|  | 112 | 112 | 0 | 11.8 | nd |
|  | 114 | 114 | 0 | 5.2 | nd |
|  | 124 | 116 | 8 | <1.5 | nd |
|  | 124 | 115 | 9 | <1.5 | nd |
|  | 124 | 115 | 9 | <1.5 | nd |
|  | 126 | 115 | 11 | <1.5 | nd |
|  | 127 | 115 | 12 | <1.5 | nd |
|  | 131 | 116 | 15 | nd | nd |
|  | 132 | 114 | 18 | <1.5 | nd |
|  | 134 | 115 | 19 | <1.5 | nd |
|  | 134 | 116 | 18 | <1.5 | nd |
|  | 134 | 115 | 19 | <1.5 | nd |
|  | 135 | 114 | 21 | <1.5 | nd |
|  | 136 | 115 | 21 | <1.5 | nd |
|  | 136 | 116 | 20 | <1.5 | nd |
|  | 140 | 117 | 23 | <1.5 | nd |
|  | 150 | 116 | 34 | <1.5 | <0.15 |
|  | 155 | 115 | 40 | <1.5 | nd |
| 37_L-63_ | 117 | 115 | 2 | <1.5 | nd |
|  | 119 | 114 | 5 | <1.5 | nd |
|  | 124 | 115 | 9 | <1.5 | nd |
|  | 126 | 115 | 11 | <1.5 | nd |
|  | 127 | 115 | 12 | <1.5 | nd |
|  | 129 | 117 | 12 | <1.5 | nd |
|  | 130 | 116 | 14 | <1.5 | nd |
|  | 131 | 116 | 15 | <1.5 | nd |
|  | 131 | 116 | 15 | <1.5 | nd |
|  | 132 | 113 | 19 | <1.5 | nd |
|  | 132 | 116 | 16 | <1.5 | nd |
|  | 132 | 115 | 17 | <1.5 | nd |
|  | 133 | 115 | 18 | <1.5 | nd |
|  | 134 | 115 | 19 | <1.5 | nd |
|  | 134 | 115 | 19 | <1.5 | nd |
|  | 135 | 115 | 20 | <1.5 | nd |
|  | 136 | 115 | 21 | <1.5 | nd |
|  | 138 | 115 | 23 | <1.5 | <0.15 |
|  | 139 | 114 | 25 | <1.5 | <0.15 |
|  | 141 | 113 | 28 | <1.5 | nd |
|  | 141 | 116 | 25 | <1.5 | nd |
|  | 143 | 113 | 30 | <1.5 | nd |
|  | 148 | 115 | 33 | <1.5 | nd |

^a^ Not determined (nd)

Table S7. HuMab concentration in spinal cord of IP dosed hSOD1-G93A mice.

| **mAb** | **age (days) at disease endpoint** | **ng of HuMab per mg of total protein in spinal cord lysate** |
| --- | --- | --- |
| IR | 118 | 40.6 |
| IR | 139 | 20.0 |
| 37_L-63_ | 118 | 2.1 |
| 37_L-63_ | 115 | 5.1 |

Table S8. HuMab serum levels with ten weeks of IP dosing.

| date of sera collection | average µg/ml at 7 days post IP (n=3)^a^ |
| --- | --- |
| **10/6** | 647 |
| **10/13** | 551 |
| **10/20** | 716 |
| **10/27** | 563 |
| **11/3** | 693 |
| **11/10** | 593 |
| **11/17** | 711 |
| **11/24** | 661 |
| **12/1** | 1007 |
| **12/8** | 539 |

^a^ Normal mice were dosed every 7 days with 50 mg/kg of irrelevant HuMab for 10 weeks and sera was collected 7 days after IP injection prior to the next injection (n = 3 per time point). HuMab concentration was determined with a quantitative ELISA for human antibody.

**Table S9. HuMab concentration in normal mouse nervous tissue and sera.**

|  | **ng HuMab / mg tissue** | | | | | | **µg/ml** |
| --- | --- | --- | --- | --- | --- | --- | --- |
|  | **spinal cord** | | **brain** | | | | **sera** |
|  | **lumbar** | **cervical** | **right hem** | **left hem** | **cerebellum** | **brain stem** |  |
| IT | 13.4 | 1.9 | <1.5 | <1.5 | <1.5 | <1.5 | 51.3 |
| IP | 26.3 | 26.7 | 33.3 | 38.0 | 37.7 | 39.3 | 544.3 |
